# Medico-legal issues related to emergency physicians’ documentation in Canadian emergency departments

**DOI:** 10.1007/s43678-023-00576-1

**Published:** 2023-08-30

**Authors:** Jeffrey D. Smith, Karen Lemay, Shirley Lee, Janet Nuth, Jun Ji, Kim Montague, Gary E. Garber

**Affiliations:** 1 Canadian Medical Protective Association, Department of Safe Medical Care, Ottawa, ON Canada; 2 Faculty of Medicine, Department of Emergency Medicine, University of Ottawa, Ottawa, ON Canada; 3 Faculty of Medicine, Department of Medicine and the School of Epidemiology and Public Health, University of Ottawa, Ottawa, ON Canada; 4 Ottawa Hospital Research Institute, Clinical Epidemiology Program, Ottawa, ON Canada

**Keywords:** Documentation, Electronic medical record, Charting, Medico-legal, Emergency medicine, Patient safety, Documentation, Dossier médical électronique, Tenue de dossier, Médico-légal, Médecine d'urgence, Sécurité des patients

## Abstract

**Objectives:**

Physician documentation plays a central role in the delivery of safe patient care. It describes a physician’s clinical decision-making and supports essential communication between healthcare providers within the patient’s circle of care. Good documentation can potentially also decrease a physician’s medico-legal risk. This study provides examples of documentation issues attributed to physicians practicing emergency medicine as identified by peer experts in civil legal actions, regulatory authority complaints (College) and hospital complaints (collectively, medico-legal cases) in Canada.

**Methods:**

We conducted a descriptive study and content analysis of medico-legal cases involving emergency department physicians from a national repository at the Canadian Medical Protective Association. Cases with peer expert criticism of an emergency physician’s documentation, which were closed between 2016 and 2020, and occurred in an emergency department were included in our analysis.

**Results:**

Of the 1628 cases involving emergency medicine, our inclusion criteria identified that absent or insufficiently detailed documentation was present in 24% of cases (391/1,628). A detailed review of 20% of the cases (79/391), selected randomly, found that documentation issues were most often associated with the assessment and investigation stage of care. This pertained to documenting details of the clinical examination, relevant medical history, diagnosis, and differential diagnosis.

**Conclusions:**

For physicians practicing emergency medicine, criticism of documentation was frequently observed in medico-legal cases. Based on the findings of this study and the expert criticism related to documentation, emergency medicine physicians may consider reflecting upon their documentation of the care provided to determine if their documentation provides a clear and accurate chronicle of the care and the rationale for their clinical decisions.

**Supplementary Information:**

The online version contains supplementary material available at 10.1007/s43678-023-00576-1.

## Résumé

**Objectifs:**

La tenue des dossiers joue un rôle crucial dans la prestation de soins sécuritaires. Elle témoigne des décisions cliniques des médecins et favorise une bonne communication entre les membres des différentes professions de la santé faisant partie du cercle de soins. Une bonne tenue des dossiers peut également réduire les risques médico-légaux auxquels les médecins sont exposés. Cette étude présente certains des problèmes relevés par les médecins experts dans la tenue des dossiers de médecins d’urgence. Elle a été réalisée à partir de dossiers d’action civile, de plaintes auprès d’organismes de réglementation de la médecine (Collège) et de plaintes auprès d’hôpitaux (dossiers médico-légaux) au Canada.

**Méthodes:**

Nous avons réalisé une étude descriptive et une analyse du contenu des dossiers médico-légaux ciblant des médecins d’urgence de la base de données nationale de l’Association canadienne de protection médicale. L’analyse incluait les dossiers conclus entre 2016 et 2020 dans lesquels les expertes et experts consultés avaient émis des critiques à l’égard de la tenue des dossiers de médecins d’urgence.

**Résultats:**

D’après nos critères d’inclusion, la tenue des dossiers avait été omise ou était insuffisante dans 391 (24%) des 1 628 dossiers ciblant des médecins d’urgence. Une analyse approfondie de 20% des dossiers (79/391), choisis au hasard, a révélé que les problèmes dans la tenue des dossiers étaient le plus souvent associés aux stades d’évaluation et d’investigation des soins. Plus précisément, les renseignements concernant les examens cliniques, les antécédents médicaux, le diagnostic et le diagnostic différentiel n’avaient pas été versés aux dossiers.

**Conclusions:**

Dans les dossiers médico-légaux, les critiques formulées à l’encontre des médecins d’urgence concernaient souvent la tenue des dossiers. À la lumière des résultats de cette étude et des critiques formulées par les médecins experts quant à la tenue des dossiers, les médecins d’urgence devraient porter attention à la consignation des soins qui sont prodigués et se demander si la tenue de leurs dossiers illustre, de façon claire et précise, la chronologie des soins ainsi que les raisons motivant leurs décisions cliniques.

## Clinician’s capsule

**Table:** 

***What is known about the topic?***
Documentation is an important but time-consuming task that often competes against other patient care demands in busy emergency departments.
***What did this study ask?***
What are the most commonly deficient aspects of an emergency physician’s clinical care documentation identified in medico-legal cases?
***What did this study find?***
Documentation issues were most often related to assessment and investigation but also common is the insufficient documentation of discussions with patients, family, substitute decision-makers and other healthcare team members.
***Why does this study matter to clinicians?***
Clinicians can review the examples of documentation issues provided and assess whether their documentation is sufficiently comprehensive for those elements.

## Background

Documentation serves multiple purposes including acting as a record of the encounter, enabling the tracking of a patient’s progress over time, and communicating with team members including consultants and the primary care provider. In Canada, physicians are taught iteratively regarding documentation of their patient assessments early in medical school. As medical trainees advance in their training, they learn to document regarding other aspects relevant to patient care as outlined by the CanMEDS and Association of Faculties of Medicine of Canada over the course of undergraduate and postgraduate medical training, generally with formative feedback from supervisors. Standardization of documentation of patient encounters is, however, lacking and not routinely reviewed or assessed over the course of a trainee’s career [1, 2].

The expectations of what a physician’s documentation should contain has evolved over time such as documenting the physician’s thought process and why investigations were or were not done to support their actions should the medical record be used in legal proceedings [3]. Although professional regulatory bodies do provide policies related to documentation of clinical encounters, these generalized principles rely somewhat on physician judgment. In a study of documentation in the emergency department, opinions of senior emergency physicians were divided on how much detail should be included [4]. This variability due to interpretation of what constitutes adequate documentation has been identified as a critical issue, particularly related to documentation omissions in medico-legal cases.

This study describes the emergency physician documentation issues identified by peer experts in civil legal actions, College complaints, and hospital complaints in Canada with the objective to bring emergency physicians’ attention to the documentation issues from medico-legal cases in specific areas of emergency medicine clinical care provision. The description of documentation issues may also be used to inform development of quality improvement or education initiatives related to physician documentation.

## Methods

### Study design

The Canadian Medical Protective Association (CMPA) provides medico-legal support, advice, and education to its over 105,000 physician members, including trainees, in Canada. The CMPA also uses its medico-legal repository to conduct safe medical care research. The repository relies on physician members to voluntarily contact the CMPA and submit materials when seeking advice or support for medico-legal matters. We performed a descriptive study and content analysis of medico-legal cases supported by the CMPA that were closed between January 1, 2016 and December 31, 2020. Cases include civil legal matters, medical regulatory authority (College) complaints and hospital complaints (defined in Online Resource 1).

### Case selection

Cases were eligible for inclusion if the named emergency physician practicing in an emergency department received peer expert criticism about a documentation issue. Cases were included regardless of the outcome. Case types were stratified by civil legal actions, College complaints and hospital complaints. Using Statistical Analysis System (SAS) Enterprise Guide 8.3, a stratified random sample of 20% of cases by case type was created using the survey select procedure. Medico-legal cases assisted by the CMPA are routinely coded using the contributing factors framework [5]. An overview of how medico-legal cases were coded by the CMPA is available in Online Resource 2. Cases were identified for inclusion from the coded medico-legal cases by using SAS Enterprise Guide 8.3 to select for cases that occurred in an emergency department and contained at least one emergency physician. These cases were then filtered for cases that contained a documentation-related contributing factor. Contributing factor codes that could indicate an issue with documentation were consent, discharge, disclosure, knowledge regarding practice management including documentation, use of electronic health record systems, transcription of a report, documentation, and handover. Finally, two authors (KM and KL) confirmed that cases with relevant contributing factor codes had peer expert criticism linked to a documentation issue by an emergency physician by reading the CMPA summary of each case. In cases where the relation could not be confirmed by the CMPA summary, then the peer expert criticism was reviewed. The authors reached consensus to include or exclude cases where there was disagreement about inclusion.

### Data collection

Confidentiality for both patients and healthcare providers was ensured by de-identification of the data. Abstracted data included patient age and gender, level of harm experienced (defined in Online Resource 2), Canadian Triage Acuity Scale scores, physician specialty, and years of practice as an emergency physician. Additional details related to abstraction of these variables are provided in Online Resource 3.

### Data analysis

Two authors (KM and KL) manually reviewed peer expert opinions assessing the care of emergency medicine physicians. We conducted a directed content analysis of peer expert opinions in the random sample of cases [6]. The authors used the CMPA’s diagnostic stages of care to code the documentation issues identified by the peer expert.

The CMPA’s diagnostic stages of care is a framework used with the contributing factors framework that reflects the phases of care a patient experiences when seeking a diagnosis and the steps a healthcare professional takes to reach a diagnosis. A committee who reviewed various published models and solicited expert opinion previously developed this framework. There are three phases within the diagnostic stages of care. First, assessment and investigation, is where the patient identifies a problem and seeks medical care and an evaluation of the patient occurs through history taking, physical examination, ordering of laboratory tests and imaging, and referring or consulting with other healthcare professionals. Second, testing, processing and interpretation, is the performance, interpretation and management of test results. Lastly, management and follow-up includes follow-up on test results and referrals, and development of treatment plan, which includes consultation and referral management (process breakdowns).

The authors used an iterative approach over a subsequent 5 weeks to code the highlighted documentation issues according to CMPA’s diagnostic stages of care in collaboration with the broader research team with clinical expertise in emergency medicine. Regular meetings were held to discuss the application of the framework, including an iterative process to proposing and assessing changes and additions to the framework to more appropriately categorize the documentation issues.

The second and third stages from CMPA’s diagnostic stages of care framework were modified and two communication codes were added following deliberation during the iterative coding process. Legibility of notes was considered related to the two communication codes, but it was coded separately because a distinction could not be made to include it in one code or another. Aspects of the third stage of care, management and follow-up, which included follow-up on test results and referrals were included instead in the second stage of care, testing, processing and interpretation. The second stage was then renamed to reassessments and results of investigations. The third stage of care focused on the remaining elements of the original third stage of care, which related primarily to discharge planning, thus we renamed the third stage of care to discharge planning.

The investigators recognized a need to distinguish communication-related documentation from within the stages of care framework as a distinct code. Further, the emergency physicians’ communication-related documentation was dichotomized as occurring between patient and substitute decision-makers versus between other physicians and healthcare providers within circle of care.

### Ethics approval

Ethics approval was obtained from the Canadian ethics review panel of the Advarra Institutional Review Board (CR00389884) in compliance with Canada’s Tri-Council Policy Statement on the Ethical Conduct for Research Involving Humans (TCPS 2).

## Results

### Case selection

**Fig. 1:**
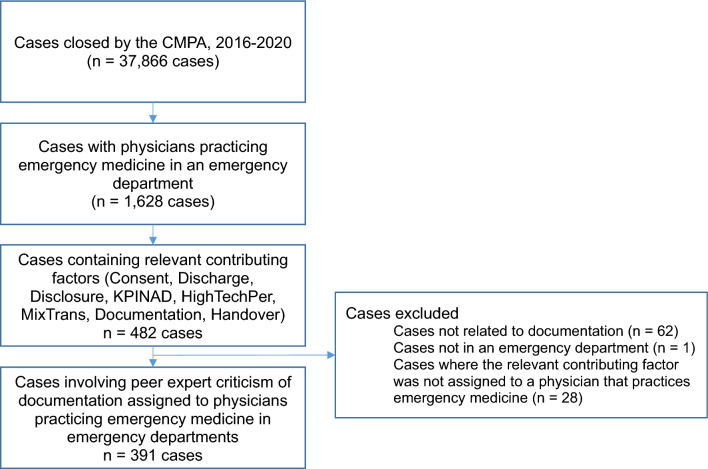
Case selection flow diagram. Relevant contributing factors were consent, discharge, disclosure, knowledge regarding practice management including documentation (KPINAD), use of electronic health record systems (HighTechPer), transcription of a report (MixTrans), documentation, or handover

The CMPA closed 37,866 medico-legal cases between 2016 and 2020. Out of 17,277 cases with medical coding, we identified 1,628 cases implicating physicians practicing emergency medicine. After manual review of cases containing a documentation-related contributing factor (*n* = 482), there were 391 cases (24% of 1628 cases) with peer expert criticism of documentation linked to a physician practicing emergency medicine in emergency departments (see Fig. 1 for the case selection flow diagram).

### Patient and physician characteristics

Details of patient and physician characteristics are included in Online Resource 4. There were 390 unique patients in 391 cases (one patient was involved in two distinct cases). There were 411 implicated physicians practicing emergency medicine in 391 cases. The number of physicians exceeds the number of cases because in 18 cases more than one physician was implicated. Thirty physicians were implicated in more than one case.

### Documentation issues

Examples of documentation issues from the peer expert opinion are summarized in Table 1 and were purposely high level to prevent identification while still providing opportunity for physicians to relate the examples to their specific clinical practice. In a detailed review of a stratified random sample of 20% of cases (*n* = 79 cases), peer expert criticism of absent or insufficiently detailed documentation was coded 117 times. Multiple documentation issues could occur in a single case and therefore the number of coded issues exceeded the number of cases. Documentation issues were most often associated with the assessment and investigation stage of care (*n* = 69), which includes the clinical examination, relevant medical history, diagnosis, and differential diagnosis (Table 1). Following the assessment and investigation category, documentation issues identified in cases in this study (in descending order of frequency), were reassessments and results of investigations (*n* = 17), communication with patient or substitute decision-makers (*n* = 14), discharge planning (*n* = 10), and communication between physicians and healthcare providers within the circle of care (*n* = 7) (Table 1). In addition to the 117 codes related to absence or insufficiently detailed documentation, illegibility was noted by peer experts in 14 (18%) of cases that were reviewed in detail.

**Table 1:** Summary of examples of peer expert criticisms of documentation with minimal details or insufficient relevant details from a seed of 20% of cases (*n* = 79)

Stage of care	Examples of clinical information that were absent or insufficiently detailed in the physician’s documentation
Assessment and investigation (*n* = 69)	Insufficient details and timing of initial assessments
	Absent positive/negative findings on history including pertinent family history/risk factors or details of the mechanism of injury
	Absent abnormal vital signs including interpretation and need for re-evaluation
	Insufficient details of the physical examination including presence/absence of pertinent findings including red flags or description of an injury or wound
	Absent differential diagnosis and rationale for decision-making, especially when excluding a more serious diagnosis
	Composite example: lack of documentation of pertinent positive and negative symptoms, details of the physical exam, and rationale for not ordering imaging to rule out cauda equina syndrome
Reassessments and results of investigations (*n* = 17)	Insufficient details and timing of subsequent assessments, patient’s response to treatment, or changes in a patient’s condition
	Absent findings of diagnostic imaging interpretation or interpretation of focused ultrasound assessment
	Absent interpretation or details of abnormal laboratory test results and need for further investigations
	Composite example: insufficient details of the neurological reassessments and rationale for cancelling diagnostic imaging in a patient with a traumatic intracranial bleed
Discharge planning (*n* = 10)	Absent discharge instructions including symptoms that should alert a patient to return to the emergency department
	Absent or insufficient details of risks and contraindications associated with a prescribed medication
	Insufficient details of discussions with patients and substitute decision-makers regarding findings, treatment plans and follow up advice
	Composite example: the discharge instructions did not include the signs and symptoms that would warrant re-evaluation and suggest a surgical etiology in a patient presenting with abdominal pain

Communication	Examples of the physician’s documentation of written and verbal communication of information that were absent or insufficiently detailed
Communication with patient or substitute decision-makers (*n* = 14)	Insufficient details of goals of care including palliative care discussions
	Absent informed consent discussions including risks and benefits, and the alternatives of a proposed procedure or treatment
	Absent informed refusal discussions including remaining overnight for observation
	Composite example: insufficient details regarding patient discussion outlining recommendations to stay for further observation regarding unresolved chest pain, and the risks to the patient in leaving the hospital before the investigations are completed to rule out acute coronary syndrome
Communication between physicians and healthcare providers within circle of care (*n* = 7)	Absent clinical information discussed during physician handover including pending patient investigations and specific reassessments required
	Absent details of pertinent healthcare team discussions including priorities for assessment
	Insufficient detail in requests for consults, out-patient referrals, or inter-facility transfers including details of critical events
	Composite example: the follow-up instructions did not alert the primary care physician to the electrolyte abnormalities that required urgent outpatient re-evaluation
Legibility (*n* = 14)	Peer experts opined the physician’s documentation was sparse and difficult to read
	Composite example: details of the physical exam that support the physician’s diagnosis were illegible in the health record

Examples are representative of frequently occurring documentation issues within each category. A total of 131 documentation issues were coded from the 79 cases reviewed. Six composites, one relating to each stage of care and categories of communication-related documentation, are provided

## Discussion

### Interpretation of findings and comparison to previous studies

This study identified where documentation issues are most commonly occurring by aligning documentation issues within a framework of the stages of care and communication-related documentation. It also included examples of the most frequent documentation issues in Canadian medico-legal cases within each component of the framework.

Peer experts commonly identified documentation deficiencies (24% of cases) in medico-legal cases assisted by the CMPA. Nearly all cases reviewed contained a documentation issue related to absence or insufficient detail pertaining to the assessment and investigation stage of care. Documentation issues related to this stage of care accounted for 53% of coded peer expert criticisms. The remaining stages of care as well as communication-related documentation were similarly prevalent to one another (range; 6–15% of coded peer expert criticisms).

A study by Walker et al. assessed quality and correctness of emergency physician documentation [7]. The authors reported 52% of documentation omissions corresponding to history taking, medical history, examination findings, and differential diagnosis [7]. These categories overlap with the assessment and investigation stage of care, which was also the stage at which omissions occurred in the majority of cases in our study.

Issues with documentation of discharge planning in an emergency department has been previously reported [8]. Cappelli and colleagues reported that 32.1% of patient charts (*n* = 117 patients) lacked sufficient detail or were missing documentation of the emergency physician’s recommendations for discharge [8].

### Strengths and limitations

The CMPA has access to a robust source of Canadian medico-legal cases involving physicians. Only a subset of cases were reviewed in detail, but stratified randomization by case type was used to reduce bias from overinclusion or underinclusion of one case type or another. The proportion of documentation issues coded to each category could change if all cases were reviewed. Nonetheless, a stratified 20% sample would likely identify the most common documentation issues. Data in this study were manually reviewed to ensure that the selected cases and data abstracted were accurate. Nonetheless, retrospective studies are limited by the accuracy of the medical and legal records available. A descriptive approach was best suited for this study because no associations could be drawn between any patient or physician variables due to a lack of an appropriate control or denominators in the data. Further, the medico-legal cases may include other areas of clinical concern unrelated to documentation and therefore, the healthcare-related harm may not be directly related to only documentation issues.

### Clinical implications

The CMPA has published good practices for documentation and record keeping as well as a reminder tool for emergency medicine physicians of frequently observed documentation issues based on analysis of CMPA medico-legal cases [9, 10]. Physicians should review and follow policies and practice standards that their relevant regulatory bodies have published (e.g., College of Physicians and Surgeons of Alberta/British Columbia/Ontario), which describes their expectations of what must be included in documentation [11–13].

### Research implications

It remains to be empirically verified whether electronic medical record implementation improves or adversely affects the quality of emergency department documentation. Future work should also examine whether copy/paste errors, use of prefilled templates, and note bloat (unnecessary, excessive documentation) contribute to medico-legal liability. We also did not identify instances of inappropriate or offensive documentation. Subsequent investigations should evaluate whether, as patients gain access to their own records, stigmatizing language in documentation becomes a source of medico-legal or regulatory complaints.

## Conclusion

This study addresses a gap in published literature of peer expert criticisms of emergency physicians’ documentation from Canadian medico-legal cases where omissions related to assessments and investigations were most common. Physicians practicing emergency medicine in Canada may reduce their medico-legal risk by reviewing the examples of documentation issues to assess whether their documentation is sufficiently comprehensive.

## Supplementary Information

Below is the link to the electronic supplementary material.

Supplementary file1 (DOCX 22 KB)

Supplementary file2 (DOCX 26 KB)

Supplementary file3 (DOCX 24 KB)

Supplementary file4 (DOCX 28 KB)

## Data Availability

Data not available due to privacy/ethical restrictions.
