# Supplementary material for: Medico-legal issues related to emergency physicians’ documentation in Canadian emergency departments
Source: CJEM. 2023 Aug 30;25(9):768–75. doi: 10.1007/s43678-023-00576-1 (PMC10495505; doi:10.1007/s43678-023-00576-1)
Supplement: Supplementary file 1 — Supplementary file1 (DOCX 22 KB) [file 43678_2023_576_MOESM1_ESM.docx]

**Online Resource 1: Medico-legal case types**

Supplemental Caption: Definition of medico-legal case types.

In the current study, each closed case represented a civil legal, College, or hospital matter defined as follows:

- Civil legal action: A physician was served or received a claim from the plaintiff or third party claimant or a defence was filed on their behalf [1].
- Civil legal threat: The CMPA referred a physician to legal counsel because there was a probability that civil litigation would be advanced against the physician [1].
- College complaint: A complaint was lodged against a physician to a medical licensing (regulatory) authority [1].
- College disciplinary matter: The matter was considered by a committee of a medical licensing (regulatory) authority whose function it was to discipline.
- College preliminary matter: An investigation, peer assessment, professional inspection, or request for personal information was commenced by a medical licensing (regulatory) authority. (These matters were distinct from those undertaken by a fitness committee, complaints committee, or discipline committee.)
- Hospital complaint: A complaint regarding a patient was lodged against a member, to a hospital or Health Authority.

Some cases may have multiple case types for the same issue. In this case the most serious case type was used (legal > College > hospital, in order of decreasing severity).

1. Calder LA, Whyte EM, Neilson HK, Zhang C, Barry TK, Barry SP. Trends and contributing factors in medico-legal cases involving spine surgery. Spine. 2022; 47(11):E469-76. Available from: <https://doi.org/10.1097/BRS.0000000000004332>.
