# Supplementary material for: Medico-legal issues related to emergency physicians’ documentation in Canadian emergency departments
Source: CJEM. 2023 Aug 30;25(9):768–75. doi: 10.1007/s43678-023-00576-1 (PMC10495505; doi:10.1007/s43678-023-00576-1)
Supplement: Supplementary file 2 — Supplementary file2 (DOCX 26 KB) [file 43678_2023_576_MOESM2_ESM.docx]

**Online Resource 2: Data capture methods at the CMPA**

Supplemental Caption: Description of data capture methods at the CMPA.

The methods for routine medical coding of closed cases at the CMPA are described below. A case is defined by the CMPA as closed when the court, College, or hospital determines a final medico-legal outcome or when there is mutual agreement between the parties to resolve the action.

Medical analysts at the CMPA are registered nurses who are trained to abstract and code medico-legal records based on CMPA procedures. Each closed case is eligible for coding if it meets appropriate criteria (e.g., included a patient, was assisted by the CMPA, and was a case type defined in Online Resource 1). As part of the medical coding process, a medical analyst writes a brief summary (the CMPA summary) in a free-text field that includes a clinical narrative, the complaint, and the medico-legal findings. Summaries and coding are restricted to physician(s) who were implicated in the case. From the named physicians in a case, the CMPA identifies implicated physicians as the physicians most involved in the issue that was central to the medico-legal case. Additional coding includes clinical variables such as the CMPA’s classification of patient harm (see Supplement 2 Table 1 below) and Canadian Triage Acuity Scale scores, if available. Medical analysts also code contributing factors as described previously [1]. In brief, criticisms from peer experts in the case are represented as a thematic code that can be classified as a provider, team or system factor. The CMPA defines peer experts as physicians who are qualified to review and interpret the issues and quality of care associated with an alleged patient complaint; most are physicians with similar training and experience as the physician named in the case [2]. In College and hospital matters, the peer experts are a College or hospital review committee who sometimes may seek advice of an expert physician to inform their decision [2]. Medical analysts also add thematic codes under teams and systems factors. Quality assurance occurs on a regular basis to reduce misclassification of case codes.

Supplement 2 Table 1. Outline of the CMPA’s definitions of patient harm of relevance to this study.^a^ Definitions were adapted from the American Society for Healthcare Risk Management’s Healthcare Associated Preventable Harm Classification Tool^b^ unless otherwise indicated.

| **Term** | **Description** |
| --- | --- |
| Patient safety incident^c^ | An event or circumstance that could have resulted, or did result, in unnecessary harm to the patient. |
| Healthcare-related harm^c^ | Harm arising from or associated with plans or actions taken during the provision of healthcare, rather than an underlying disease or injury. |
| No harm (Asymptomatic) | Patient safety event or patient safety incident that reached the patient but the patient reports no symptoms and no treatment is required. |
| Mild harm | Patient harm is symptomatic, symptoms are mild, loss of function or harm is minimal (permanent or temporary), and minimal or no intervention is required (e.g., extra observation, investigation, review, or minor treatment). |
| Moderate harm | Patient harm is symptomatic, requiring intervention (e.g., additional moderate or minor operative procedure, additional therapeutic treatment), or an increased length of stay, or causing permanent or temporary harm, or loss of function. |
| Severe harm | Patient harm is symptomatic, requiring life-saving intervention or major medical/surgical intervention, or resulting in a shortening life expectancy, or causing major permanent or temporary harm or loss of function. |
| Death | Healthcare-related death |

^a^ Calder LA, Neilson HK, Whyte EM, Ji J, Bhatia RS. Medico-legal cases involving cardiologists and cardiac test underuse or overuse. CJC open. 2021 Apr 1;3(4):434-41. Available from: https://doi.org/10.1016/j.cjco.2020.11.018

^b^ Hoppes M, Mitchell J. Serious safety events: A focus on Harm Classification: Deviation in care as link. Getting to Zero^TM^ White Paper Series Edition No. 2 ed. Chicago, Illinois: American Society for Healthcare Risk Management, 2014.

^c^ World Health Organization. More than Words: Conceptual Framework for the International Classification for Patient Safety - Final Technical Report. World Health Organization; 2009.

1. McCleery A, Devenny K, Ogilby C, et al. Using medicolegal data to support safe medical care: A contributing factor coding framework. J Healthc Risk Manag 2019;38(4):11-8.
2. Calder LA, Whyte EM, Neilson HK, Zhang C, Barry TK, Barry SP. Trends and Contributing Factors in Medico-legal Cases Involving Spine Surgery. Spine. 2022; 47(11):E469-76. Available from: <https://doi.org/10.1097/brs.0000000000004332>.
