# Supplementary material for: Medico-legal issues related to emergency physicians’ documentation in Canadian emergency departments
Source: CJEM. 2023 Aug 30;25(9):768–75. doi: 10.1007/s43678-023-00576-1 (PMC10495505; doi:10.1007/s43678-023-00576-1)
Supplement: Supplementary file 3 — Supplementary file3 (DOCX 24 KB) [file 43678_2023_576_MOESM3_ESM.docx]

**Online Resource 3: Detailed Methods**

Supplemental Caption: Additional details of study methods.

*Data analysis*

Confidentiality for both patients and healthcare providers was achieved by de-identification of the data. Frequencies were calculated using SAS directly from the repository for patient demographics and disease variables, and physician specialty. For physicians named in multiple cases, frequencies represent the physicians’ characteristics at the date of occurrence of when care was provided to the patient

We derived years practicing in Canada as a physician with emergency specialty based on the date of occurrence of the medico-legal event and the CMPA’s membership data, which includes self-reported physician specialty for each year of CMPA membership. The CMPA has collected specific specialty member data since 1984 for administrative purposes when obtaining CMPA membership, which allows estimation of years of practice as an emergency physician. Years of practice outside of Canada and when the physician was not a member of the CMPA are not included.
