# Supplementary material for: Medico-legal issues related to emergency physicians’ documentation in Canadian emergency departments
Source: CJEM. 2023 Aug 30;25(9):768–75. doi: 10.1007/s43678-023-00576-1 (PMC10495505; doi:10.1007/s43678-023-00576-1)
Supplement: Supplementary file 4 — Supplementary file4 (DOCX 28 KB) [file 43678_2023_576_MOESM4_ESM.docx]

**Online Resource 4 – Additional Tables**

Supplemental Caption: Additional tables

Supplement 4 Table 1. Patient characteristics in CMPA medico-legal cases (closed 2016-2020) with peer expert criticism of a documentation issue linked to a physician practicing emergency medicine, N = 391 patients^a^.

| Characteristics | No. (%) of patients |
| --- | --- |
| **Demographic** |  |
| Age (yr), median (IQR)^b^ | 48 (32-65) |
| Age (yr) |  |
| < 2 | 8 (2.0) |
| ≥ 2 to 17 | 30 (7.7) |
| ≥ 18 to 29 | 38 (9.7) |
| ≥ 30 to 64 | 204 (52.2) |
| ≥ 65 to 79 | 59 (15.1) |
| ≥ 80 | 37 (9.5) |
| Unknown | 15 (3.8) |
| Gender |  |
| Female | 208 (53.2) |
| Male | 177 (45.3) |
| Unknown | 6 (1.5) |
| **Disease** |  |
| Level of harm^c^ |  |
| None (asymptomatic) | 48 (12.3) |
| Mild | 57 (14.6) |
| Moderate | 114 (29.2) |
| Severe | 24 (6.1) |
| Death | 81 (20.7) |
| Not applicable, unknown, or a near miss | 67 (17.1) |
| Canadian Triage Acuity Scale^d^ |  |
| Level 1 – Resuscitation | 5 (1.3) |
| Level 2 – Emergent | 61 (15.6) |
| Level 3 – Urgent | 113 (28.9) |
| Level 4 – Less urgent | 56 (14.3) |
| Level 5 – Non-urgent | 15 (3.8) |
| Unknown | 128 (32.7) |

^a^ One patient was involved in two distinct medico-legal cases.
^b^ Based on 376 patients with known age.
^c^ Based on the CMPA’s classification of patient harm (Online Resource 2). Harm is the healthcare-related harm arising from (or associated with) the plans or actions taken during the provision of healthcare, rather than an underlying disease or injury (World Health Organization. More than Words: Conceptual Framework for the international Classification for Patient Safety - Final Technical Report. 2009. Available from: https://www.who.int/publications/i/item/WHO-IER-PSP-2010.2).
^d^ Canadian Triage Acuity Scale tool is available from: <https://ctas-phctas.ca/>

Supplement 4 Table 2. Characteristics of physicians practicing emergency medicine in an emergency department in CMPA medico-legal cases (closed 2016-2020) with peer expert criticism of a documentation issue, N = 411 physicians^a,b^.

| Characteristics | No. (%) of physicians |
| --- | --- |
| **Specialty of physician** |  |
| Emergency physician | 300 (73.0) |
| Family physician/general practice with shifts in emergency department | 111 (27.0) |
| **Years practicing in Canada as a physician with emergency specialty^c^** |  |
| <5 | 105 (25.5) |
| 6-10 | 82 (20.0) |
| 11-20 | 93 (22.6) |
| 21-30 | 77 (18.7) |
| 30+ | 54 (13.1) |

^a^ The number of physicians exceeds the number of cases because 18 cases involved more than one physician practicing emergency medicine with peer expert criticism of a documentation issue linked (17 cases with two implicated physicians, one case with four implicated physicians).
^b^ For physicians named in multiple unique cases, frequencies represent the physicians’ characteristics at the date of occurrence of when care was provided to the patient (30 physicians were involved in more than one case).
^c^ Calculated as years of CMPA membership after residency.
